# Stability of plant virus-based nanocarriers in gastrointestinal fluids†
†Electronic supplementary information (ESI) available. See DOI: 10.1039/c7nr07182e


**DOI:** 10.1039/c7nr07182e

**Published:** 2017-12-05

**Authors:** Alberto Berardi, David J. Evans, Francesca Baldelli Bombelli, George P. Lomonossoff

**Affiliations:** a Department of Pharmaceutical Sciences and Pharmaceutics , Faculty of Pharmacy , Applied Science Private University , Amman 11931 , Jordan . Email: a_berardi@asu.edu.jo ; Fax: (+962)65515017 ; Tel: +9626 5609999; b Department of Biological Chemistry , John Innes Centre , Norwich Research Park , Norwich , NR4 7UH , UK; c Laboratory of Supramolecular and BioNano Materials (SupraBioNanoLab) , Department of Chemistry , Materials and Chemical Engineering , Politecnico di Milano , Milano , Italy

## Abstract

Cowpea mosaic virus (CPMV) is a plant virus which is being extensively investigated as a drug delivery and vaccine nanocarrier for parenteral administration. However, to date little is known about the suitability of plant-based nanocarriers for oral delivery. In this study, the colloidal (*i.e.* aggregation), physical (*i.e.* denaturation) and chemical (*i.e.* digestion of the polypeptides) stability of CPMV and its empty virus-like particles (eVLPs) in conditions resembling the gastrointestinal fluids were evaluated. The nanoparticles were incubated in various simulated gastric and intestinal fluids and in pig gastric and intestinal fluids. CPMV and eVLPs had similar stabilities. In simulated gastric media, they were stable at pH ≥ 2.5. At lower pH destabilisation of the particle structure occurred, which, in turn, rendered the polypeptides extremely sensitive to pepsin digestion. However, both CPMV and eVLPs were stable in simulated intestinal fluids, in pig gastric fluids and in pig intestinal fluids. Thus CPMV, despite being a protein-based nanoparticle, was much more resistant to the harsh GI conditions than soluble proteins. Remarkably, both CPMV and eVLPs incubated in pig gastric and intestinal fluids were not subject to protein adsorption, with no formation of a detectable protein corona. The lack of a protein corona on CPMV and eVLP surfaces in GI fluids would imply that, if orally administered, these nanoparticles could maintain their native surface characteristics; thus, their biological interactions would remain predictable and unchanged. In summary, CPMV and eVLPs can be considered promising nanocarriers for applications requiring oral delivery, given their chemical, physical and colloidal stability and lack of protein adsorption from the environment in most of the tested conditions.

## Abstract 2

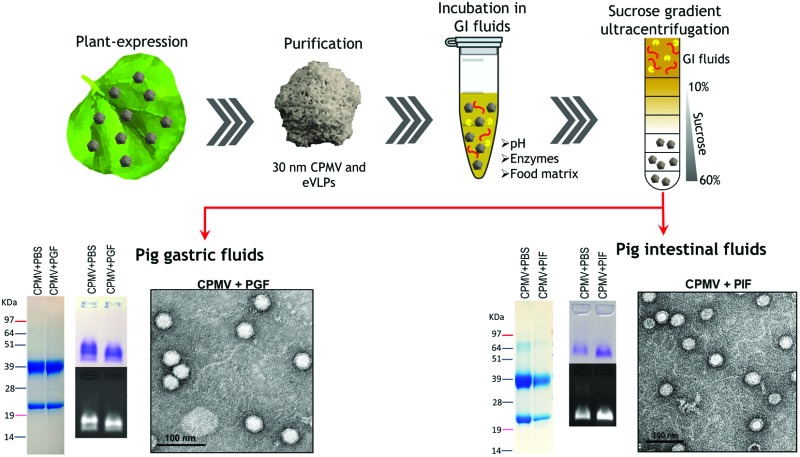
Viral nanoparticles in gastric and intestinal fluids: evaluation of digestion, denaturation, aggregation and protein corona formation.

## 1. Introduction

In the last few years, there has been growing interest in the use of naturally-derived nanocarriers for drug delivery and bio-imaging. Viral nanoparticles (VNPs) constitute one important class of “bionspired” nanosystems.^1^ VNPs are generally plant viruses or virus-like particles (VLPs): plant viruses consist of proteinaceous shells containing the nucleic acids necessary for the viral replication in plants; however plant viruses cannot infect humans. Whereas, VLPs are viral mimics that are non-infectious not only to humans, but also to plants. VNPs are promising carriers for drug and vaccine delivery: being well-defined gene products, they have ordered architectures and are monodisperse. Their hollow protein shell allows encapsulation of high concentrations of therapeutics. Also, VNPs are biocompatible, biodegradable and can be mass produced. Finally, VNPs can be genetically and chemically modified on their protein subunits to provide additional functionalities to the nanoparticles (including cell-specific targeting).^2^

Cowpea mosaic virus (CPMV) is a plant picornavirus with icosahedral shape and a diameter of 30 nm. The capsids are made up of 60 copies each of a small (S) and large (L) proteins, naturally encapsulating two positive sense RNA molecules.^3^ CPMV, and its empty virus-like particles (eVLPs),^4^ have been extensively studied for use in nanomedicine: it is known that CPMV selectively binds vimentin receptors, which are over-expressed on the surface of endothelial and cancer tissues, potentially enabling the delivery of bio-imaging agents or anticancer agents to those tissues.^5^,^6^ Both CPMV^7^,^8^ and eVLPs^9^ have been loaded with various compounds, including cytotoxic agents. CPMV has also been used in vaccinology as a scaffold for the display of heterologous immunogenic epitopes against animal pathogens.^10^ eVLPs have not only been use as drug and vaccine carriers, but also as cancer immunotherapy agents.^11^

To date, most studies on the use of VNPs in medicine have focused on parenteral administration. Given the great potential that CPMV holds as a drug delivery and vaccine platform, it is important to determine whether this nanocarrier could also be exploited for oral drug or vaccine delivery. The use of nanomaterials to improve the oral bioavailability of anticancer drugs and therapeutic peptides and proteins (including vaccines) is an area of extensive research.^12^ However, the oral delivery of nanoparticles (NPs) is challenging, due to the inherently unfavourable physio-chemical properties of the gastrointestinal (GI) tract: the extremely variable range of pH, the high ionic strength, the presence of digestive enzymes and the presence of a food matrix, all constitute barriers to the stability and bioavailability of the loaded nanomaterials.^13^–^15^ In particular, all these factors influence the colloidal stability of the NPs leading to extensive agglomeration.^16^–^18^ Moreover, it is known that the exposure of NPs to biological fluids often results in the formation of a “protein corona” surrounding the NP, which not only can greatly affect their size, but also their surface properties and thus biochemical response.^19^ It has been recently pointed out that while most studies focus on the formation of the protein corona in blood, this phenomenon is also expected to occur in the GI tract, yet this has been only minimally investigated.^20^–^22^ It must be emphasised that VNPs are protein-based NPs. Therefore, if orally administered, their stability could be also compromised by physical denaturation of their quaternary structure at gastric pH and possible proteolysis by digestive enzymes.^23^

Little is currently known about the fate of orally administered CPMV. It has been shown that L and S protein of CPMV were not digested when incubated at a fixed concentration of pepsin and pancreatin.^24^ Also, when orally administered to mice, native CPMV could reach the systemic circulation, though the amount of measurable CPMV accumulated in the spleen was 10–100 fold lower than when CPMV was administered by injection.^24^,^25^ Despite this preliminary information, little is known about the susceptibility of CPMV to the harsh and heterogeneous conditions of the human gastric and intestinal fluids, in terms of both colloidal stability and formation of a protein corona. To address these aspects, we have exposed both CPMV and eVLPs to different *in vitro* media simulating the GI conditions as well as to pig gastric (PGF) and intestinal fluids (PIF). Due to ethical limitations on retrieving human GI fluids, PGF and PIF were chosen as *ex vivo* media, based on recent findings that indicate they can be used as surrogate of human GI fluids in the evaluation of the stability of biopharmaceuticals.^26^,^27^

Here, we investigate: (1) the chemical stability of the CPMV L and S proteins when VNPs are exposed to conditions reflecting the GI environment and in pig GI fluids; (2) the tendency to denaturation (*i.e.* physical stability at the level of single particles) of CPMV and eVLPs upon exposure to artificial and pig GI fluids; (3) the tendency to aggregation (*i.e.* physical stability at macroscopic level) of CPMV and eVLPs upon exposure to artificial and pig GI fluids; and (4) the formation of a protein corona upon exposure of CPMV and eVLPs to the complex matrix of the pig GI fluids.

## 2. Experimental section

### 2.1. Materials

pBinPS1NT and pBinPS2NT were used for the production of infectious CPMV in plants^28^ while pEAQexpress-VP60-24 K was used for the expression of eVLPs.^4^,^29^ Complete® Protease inhibitor tablets were bought from Roche (UK). 12% (w/v) NuPAGE polyacrylamide bis–tris gels, NuPAGE MOPS buffer, NuPAGE LDS Sample Buffer were obtained from Invitrogen (UK). Brilliant Blue R Concentrate and InstantBlue stain were purchased from Sigma (UK) and Expedon (UK), respectively. Amicon centrifugal filters (100 kDa MWCO) and Micro Float-A-Lyzer dialysis tube (100 kDa MWCO) were purchased from Millipore and Spectrum Laboratories, respectively. Bovine Serum Albumin (BSA, heat shock fraction), pepsin (≥400 units per mg protein) from porcine gastric mucosa and pancreatin (≥3 × USP specifications) from porcine pancreas were obtained from Sigma-Aldrich (UK). Three full-length gastrointestinal tracts were procured from freshly killed pigs at a local abattoir (H. G. Blake Costessey Ltd, Norwich, UK). The animals were processed under standard UK legislation for food-producing animals, the gut extracted within less than an hour of slaughter and transported intact to the laboratory on ice.

A summary of the composition of the media used in this study is presented in Table 1.

**Table 1:** Composition of the simulated and gastric and intestinal fluids used in the study

Media	Name	Composition
Simulated gastric fluids without enzymes	pH 1	100 mM HCl + 34 mM NaCl
	pH 1.2	63 mM HCl + 34 mM NaCl (British Pharmacopoeia, 2012)
	pH 1.5	32 mM HCl + 34 mM NaCl
	pH 2	10 mM HCl + 34 mM NaCl
	pH 2.5	3.2 mM HCl + 34 mM NaCl
	pH 3	1 mM HCl + 34 mM NaCl
	pH 3.5	0.32 mM HCl + 34 mM NaCl
	pH 4	0.1 mM HCl + 34 mM NaCl
Simulated gastric fluids with enzymes	SGF-pH 1	100 mM HCl + 34 mM NaCl + 0.32% (w/v) pepsin
	SGF-pH 1.2	63 mM HCl + 34 mM NaCl + 0.32% (w/v) pepsin (British Pharmacopoeia, 2012)
	SGF-pH 1.5	32 mM HCl + 34 mM NaCl + 0.32% (w/v) pepsin
	SGF-pH 2	10 mM HCl + 34 mM NaCl + 0.32% (w/v) pepsin
	SGF-pH 2.5	3.2 mM HCl + 34 mM NaCl + 0.32% (w/v) pepsin
	SGF-pH 3	1 mM HCl + 34 mM NaCl
	SGF-pH 3.5	0.32 mM HCl + 34 mM NaCl + 0.32% (w/v) pepsin
	SGF-pH 4	0.1 mM HCl + 34 mM NaCl + 0.32% (w/v) pepsin
Simulated intestinal fluid	SIF	50 mM KH_2_PO_4_ (+NaOH to pH 6.8) + 1% (w/v) pancreatin (British Pharmacopoeia, 2012)
Pig gastric fluid	PGF	pH 3.9 (±0.1)^*a*^
Pig intestinal fluid	PIF	pH 6.3 (±0.2)^*a*^

^*a*^Mean (±S.D.), *n* = 3.

### 2.2. General methods

#### 2.2.1. CPMV and eVLPs expression and purification

Infectious CPMV and eVLPs were extracted from leaves 6–7 days post-infiltration and purified according to previously established methods.^29^ Purified CPMV and eVLPs were stored in water or PBS at 4 °C.

#### 2.2.2. Measurement of CPMV and eVLPs concentration by UV absorbance

CPMV concentration in purified samples was spectrophotometrically determined by measurement of the absorbance at a wavelength of 260 nm (A260), using a NanoDrop Spectrophotometer ND-1000. Sample concentration was calculated from the molar extinction coefficient of CPMV at the same wavelength (molar extinction coefficient at 260 nm *ε* = 8.1 mL mg^–1^ cm^–1^).^30^ Purified eVLPs were quantified by measurement of the absorbance at 280 nm (A280 – molar extinction coefficient at 280 nm *ε* = 1.28 mL mg^–1^ cm^–1^).^29^

For semi-quantitative measurements of protein distribution in sucrose gradients, the A280 of the fractions was measured.

#### 2.2.3. Electrophoresis methods

For Sodium Dodecyl Sulfate Polyacrylamide Gel Electrophoresis (SDS-PAGE), protein samples were denatured by adding one volume of LDS loading buffer to two volumes of the samples. Then 10–20 μL of each sample were separated on 12% (w/v) NuPAGE polyacrylamide gels in 1× MOPS running buffer at 200 V for 50 min. The gels were stained with Instant Blue.

For native agarose gel electrophoresis, undenatured samples of CPMV and eVLPs (generally 10 μg) were analysed by agarose gel electrophoresis. Gels [1.2% (w/v) agarose in Tris/Borate/EDTA (3.03 g L^–1^ Tris-HCl, 5.5 g L^–1^ boric acid, 2 mM EDTA, pH 8.3)] were run at 60 V for 90 min and were then stained with Brilliant Blue R Concentrate for 30 min. In the case of samples containing CPMV, another gel was run in parallel and stained with a 0.5 mg mL^–1^ solution of ethidium bromide for 30 min and then visualised under UV light (wavelength = 302 nm).^31^

#### 2.2.4. DLS measurements

Dynamic Light Scattering (DLS) was measured using a DynaPro Titan, Wyatt Technology Corporation (laser wavelength 830 nm, scattering angle 20°) and Dynamics software. 13 μL samples of 0.2 mg mL^–1^ of VNPs were analysed at 25 °C. Data were presented as an average of three measurements of 10 runs each.^30^

#### 2.2.5. Transmission electron microscopy (TEM)

Samples at a concentration of CPMV or eVLPs of approximately 0.1–0.3 mg mL^–1^ were adsorbed onto hexagonal, plastic and carbon-coated copper grids. The grids were negatively stained with 2% (w/v) uranyl acetate before being imaged using a FEI Tecnai G2 20 Twin TEM with a built-in digital camera. Particle diameter was calculated as the average of 25 particles for each TEM image as measured using ImageJ software.

### 2.3. CPMV and eVLPs stability in simulated gastric fluid without pepsin

The stability of CPMV and eVLPs in simulated gastric fluid without pepsin was evaluated by sucrose density gradient ultracentrifugation, native agarose gel, TEM and DLS.

For sucrose gradient ultracentrifugation, 0.25 mL of 6 mg mL^–1^ purified CPMV or eVLPs were incubated for two hours with 3.75 mL of a pH 1.2 solution (Table 1). After the incubation, the preparations were loaded on the top of pH 1.2 10–60% sucrose gradients. Alternatively, CPMV or eVLPs preparations were identically incubated at pH 1.2 for two hours and then the pH was neutralised by addition of 1.2 mL of 400 mM NaHCO_3_. The neutralised samples were layered on the top of 10–60% sucrose gradients prepared in phosphate buffer saline pH 7.4 (PBS). As control, identical CPMV or eVLPs preparations were incubated in PBS and layered on the top of a 10–60% sucrose gradient in PBS. The gradients were centrifuged at 31 200 rpm for 2.5 hours at 4 °C in a TH-641 rotor.^23^ Following centrifugation, fractions were collected from the gradients, and analysed by SDS-PAGE and by UV spectrophotometry.

For native agarose gels, 6 mg mL^–1^ CPMV or eVLPs in water were diluted 1 : 9 in either PBS (control), pH 1, pH 1.5, pH 2, pH 2.5, pH 3, pH 3.5 or pH 4 (Table 1). After two hours incubation, the eight samples were loaded on agarose gel for electrophoresis.^23^

For TEM analysis, 6 mg mL^–1^ CPMV or eVLPs in water were diluted 1 : 19 in either PBS (control) or at pH 1.2 (Table 1). After two hours incubation the sample was used for imaging. In addition, one sample of CPMV incubated at pH 1.2 was neutralised with 400 mM NaHCO_3_ prior to TEM.^23^

For DLS measurements, 6 mg mL^–1^ CPMV preparations in water were diluted 1 : 24 in either PBS (control), pH 1.2, pH 2, pH 3 or pH 4 solutions (Table 1), which had been previously passed through 0.22 μm filters. After two hours incubation, the samples were analysed by DLS. Alternatively, CPMV preparations were identically incubated at the aforementioned different pH; after two hours the pH was neutralised by addition of 1 M Tris-HCl pH 8 (in a ratio of 1 : 5) and the resulting samples were characterised by DLS. Tris-HCl was chosen as neutralising agent rather than bicarbonate, as the latter base reacts with acids, resulting in the formation of gas in solution, which could interfere with the DLS analysis.

### 2.4. CPMV and eVLPs stability in simulated gastric fluids with pepsin (SGF)

The stability of CPMV and eVLPs in simulated gastric fluid with pepsin was evaluated by SDS-PAGE and native agarose gel.

6 mg mL^–1^ of CPMV or eVLPs in water were diluted 1 : 9 in different pH 1.2 solutions containing 10% (w/v), 3.2%, 1%, 0.32% (SGF-pH 1.2 – Table 1), 0.1%, 0.032, 0.01% pepsin.^23^ As a control, bovine serum albumin (BSA) was incubated in a pH 1.2 solution containing 32 mg mL^–1^ pepsin. After two hours incubation at 37 °C, the samples were boiled to stop the enzymatic reaction and loaded on SDS-PAGE gels. Experiments were also performed diluting CPMV or eVLPs in pH 3 solutions with an identical range of pepsin concentrations as the one mentioned above.

For native agarose gels, 6 mg mL^–1^ of CPMV or eVLPs in water were diluted 1 : 9 in PBS (control), SGF-pH 1, SGF-pH 1.5, SGF-pH 2, SGF-pH 2.5, SGF-pH 3, SGF-pH 3.5 or SGF-pH 4 (Table 1), each media containing a fixed concentration of enzyme (0.32% pepsin^32^).

### 2.5. CPMV and eVLPs stability in simulated intestinal fluids (SIF)

Simulated intestinal fluid (SIF) (Table 1) was prepared according to British Pharmacopoeia.^32^ Six aliquots of 6 mg mL^–1^ CPMV or eVLPs in water were diluted 1 : 9 in SIF and incubated at 37 °C for 4, 3, 2, 1, 0.5 and 0.25 hours. As a control, bovine serum albumin (BSA) was incubated in SIF for 4 hours. After the incubation each sample was diluted 1 : 9 in a 10× protease inhibitor solution in water and analysed by SDS-PAGE and native agarose gels.^23^

### 2.6. Preparation of pig gastric fluids (PGF) and pig intestinal fluids (PIF)

Three full-length gastrointestinal tracts, collected from freshly killed animals and immediately transported to the laboratory, were dissected. Gastric and intestinal fluids from the upper small intestine were collected within three hours from sacrifice and immediately stored at –80 °C until use. Prior to stability studies, frozen aliquots were thawed and the solid content was pelleted by centrifugation at 9300*g* for 10 min^23^,^27^ The supernatant was used as PGF and PIF in the following stability studies. The mean pH of PGF and PIF is presented in Table 1.

### 2.7. CPMV and eVLPs stability in pig gastric fluids (PGF) and intestinal fluids (PIF)

6 mg mL^–1^ CPMV in water was diluted 1 : 9 with PGF or PIF and incubated at 37 °C for two hours or four hours, respectively. As negative control, PGF or PIF were pre-boiled at 98 °C for 10 minutes to inactivate the enzymes; CPMV was then diluted 1 : 9 in the pre-boiled PGF or PIF. As positive control, BSA was added to pre-boiled PGF and PGF or pre-boiled PIF and PIF. After the incubation, all samples were boiled, diluted 1 : 1 in water and analysed by SDS-PAGE.

For sucrose gradient ultracentrifugation, 50 μL of 6 mg mL^–1^ purified CPMV were incubated with 450 μL PGF or PIF at 37 °C for two hours or four hours, respectively. Then, 150 μL of 400 mM NaHCO_3_ or 55 μL of 10× protease inhibitor solution were added to the PGF preparations^33^ or PIF preparation,^23^ respectively, to stop the enzymatic activity. The samples were diluted to 2.4 mL and loaded on the top of 10–60% sucrose gradients in PBS. As positive control, CPMV was incubated in PBS and layered on identical sucrose gradients. As a negative control, 450 μL PGF or PIF were diluted with 50 μL water and loaded on sucrose gradient after the 2 hours incubation. The ultracentrifugation was run for 2.5 hours at 4 °C at 37 500 rpm in a AH-650 rotor.^23^ Fractions from the gradients were collected, analysed by SDS-PAGE and A280 UV spectrophotometry. Experiments were performed in triplicates.

Sucrose fractions that were found to contain CPMV from the incubation in PGF or PIF were also pooled together, dialysed against water and concentrated to approximately 0.5 mg mL^–1^ of protein using centrifugal filters.^22^ The resulting samples were analysed by SDS-PAGE, native agarose gel and TEM.

Identical experiments were carried out for eVLPs, with the exception of the UV analysis.

## 3. Results and discussion

### 3.1 Stability of CPMV upon exposure to simulated gastric fluids (SGF) without enzymes

**Fig. 1:**
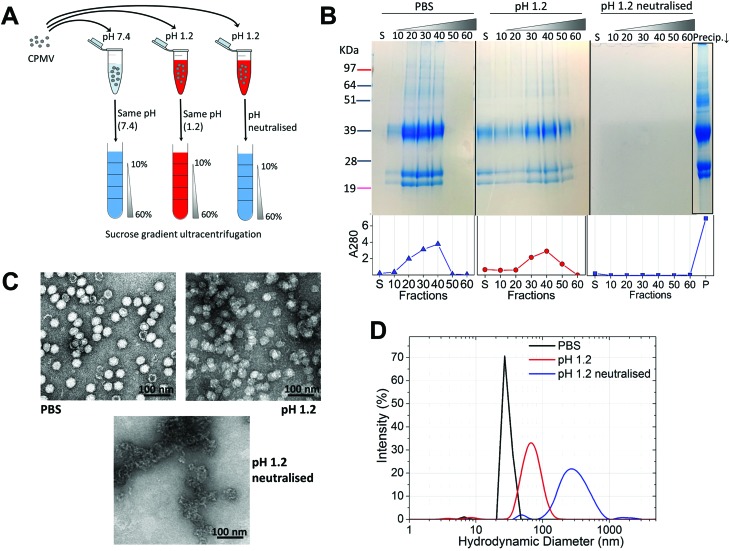
CPMV physical stability in pH 1.2. A: schematic diagram of the stability studies: the sucrose gradient ultracentrifugation was used as a means to assess the VNPs’ integrity. CPMV was incubated for two hours in either PBS (control) or pH 1.2, followed by density gradient ultracentrifugation with the sucrose dissolved in either PBS or pH 1.2, respectively. A third sample was exposed to pH 1.2, the pH neutralised after two hours and the density gradient ultracentrifugation performed in PBS. B: SDS-PAGE (top) and UV absorbance at 280 nm (bottom) of the sucrose gradient fractions, after 2 hours incubation of CPMV in PBS (control), pH 1.2 and pH 1.2 followed by pH neutralisation. S, indicates the supernatant; 10, 20, 30, 40, 50 and 60 indicate the percentages of sucrose of the collected fractions; Precip.↓ indicates the precipitate formed after the ultracentrifugation. C: TEM images of CPMV after 2 hours incubation in PBS (control), pH 1.2 and pH 1.2 followed by pH neutralisation. D: DLS of CPMV after 2 hours incubation in PBS (control), pH 1.2 and pH 1.2 followed by pH neutralisation.

Initially, the stability of CPMV was evaluated at pH 1.2 (Table 1). Fig. 1A shows the set-up of the experiment. SDS-PAGE analysis of the gradient fractions following ultracentrifugation (Fig. 1B) showed that, in the PBS control, CPMV L (39 kDa) and S protein (25 and 22 kDa) banded in the 20, 30 and 40% sucrose fractions (Fig. 1B – left), as typical of intact VNPs.^34^ In contrast, CPMV exposed to pH 1.2 was found to be spread between most fractions of the gradient (Fig. 1B – centre). Finally, when CPMV was exposed to pH 1.2 and then the pH was neutralised, the L and S proteins could not be detected in any fraction; however, a precipitate containing the L and S proteins could be found at the bottom of the centrifugation tube (Fig. 1B – right). UV absorbance of the corresponding sucrose gradient fractions (Fig. 1B – bottom) confirmed the SDS-PAGE results. For further characterisation, preparations of CPMV incubated in PBS (control), pH 1.2 or pH 1.2 followed by pH neutralisation were analysed by TEM and DLS (Fig. 1C and D, respectively). TEM showed icosahedral CPMV particles of approximately 30 nm in diameter for the control; at pH 1.2 VNPs can still be visualised, though they appear more irregular in shape and size and more aggregated; at pH 1.2 neutralised, only clusters of materials can be seen and no discrete particles (Fig. 1C). The plots of frequency distributions (Fig. 1D) and the mean hydrodynamic diameter and percentage of polydispersity (Table 2) showed a similar trend: a monodispersed population of particles with a mean diameter of 29.4 (±0.3) nm corresponding to intact CPMV was measured for the control.^7^ An evident size increase, as well as in increase in polydispersity resulted from the exposure of CPMV to pH 1.2. Upon exposure to acid and neutralisation of the pH a further size increase and a much broader distribution can be noted, indicating that CPMV had aggregated.

**Table 2:** Average hydrodynamic diameter and polydispersity of CPMV incubated for two hours with different acidic media, as determined by DLS (mean ± SD, *n* = 3)

Condition	Mean diameter (nm)	Polydispersity (%)
PBS (control)	29.4 (±0.3)	13.3 (±0.4)
pH 1.2	70.3 (±2.5)	26.5 (±6.1)
pH 2	53.3 (±1.3)	15.1 (±2.2)
pH 3^*a*^	46.9 (±6.2)	26.6 (±19.6)
pH 4	31.0 (±0.0)	14.4 (±0.4)
pH 1.2 neutralised after 2 hours	320.7 (±24.7)	36.4 (±6.9)
pH 2 neutralised after 2 hours	113.2 (±2.5)	34.9 (±3.8)
pH 3 neutralised after 2 hours	31.6 (±1.1)	13.4 (±0.6)
pH 4 neutralised after 2 hours	31.8 (±0.2)	13.6 (±0.1)

^*a*^A bimodal distribution was measured; mean and polydispersity of the main peak are presented.

These results, taken together, provide an insight into the stability of CPMV upon exposure to an acidity comparable to that of the fasted gastric stomach.^23^,^35^ The observation that CPMV could be found throughout the gradient following ultracentrifugation at pH 1.2, and not confined to the 20, 30 and 40% fractions as in the control, indicates that VNPs underwent morphological changes that affected size and/or density. Indeed, both TEM and DLS measurements confirmed that particles increased in size and formed a more heterogeneous population, compared to the control. However, despite the harsh conditions of incubation, complete disassembly of VNPs did not occur and particles were still present. By contrast, upon acidification and subsequent pH neutralisation the particulate structure of CPMV was totally lost and the L and S proteins aggregated in large clusters (Fig. 1B, C and D, respectively). These findings suggest that if CPMV were administered orally and exposed to the conditions of the fasting stomach, the pH would not itself completely disassemble the nanoparticles (NP); however, when the pH returns to near neutrality, *i.e.* in the intestine, aggregation is likely to occur. In a previous study, a similar instability pattern was found for Hepatitis B core antigen VLPs.^23^

**Fig. 2:**
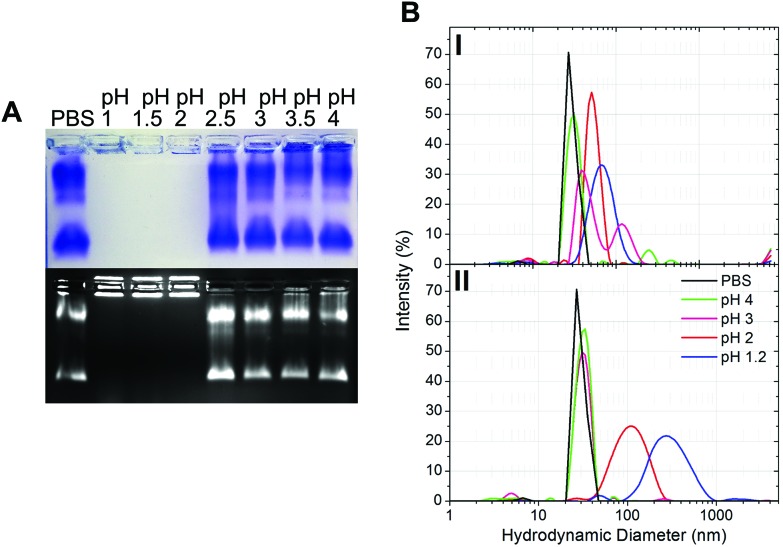
Physical stability of CPMV in simulated gastric fluids at different pH (without pepsin). A: Coomassie-stained (top) and ethidium bromide-stained (bottom) native agarose gel of CPMV after 2 hours incubation in simulated gastric fluids at different pH and in PBS (control). B: Intensity-based particle size distribution of CPMV after 2 hours incubation in simulated gastric fluids at different pH (I) and at the different pH, but followed by pH neutralisation (II), as measured by DLS; CPMV was incubated in PBS for positive control.

The gastric pH typically ranges from 1 to 2 in fasting conditions and from 3 to 7 in fed conditions.^36^ Thus, the stability of CPMV exposed to solutions of hydrochloric acid at pH 1, 1.5, 2, 2.5, 3, 3.5 and 4 was evaluated, in order to reflect the heterogeneity and the constantly changing composition of human gastric fluids. The stability of the VNPs was measured by native agarose gel electrophoresis and DLS (Fig. 2A and B, respectively). In the case of CPMV incubated for two hours in PBS (control) two bands could be seen in the both the Coomassie- and ethidium bromide-stained gels (Fig. 2A, upper and lower gel). The stained protein capsid and nucleic acid in the two gels had the same electrophoretic mobility, consistent with previous reports.^31^,^37^ The same bands as in the control can be seen for CPMV incubated at pH ≥ 2.5, indicating that VNPs remained intact. Bands corresponding to intact VNPs were not observed at pH 1, 1.5 and 2, and the nucleic acid appeared aggregated and trapped in the wells. In Fig. 1 it was shown that CPMV aggregated not upon acidification, but upon subsequent neutralisation thus it is likely that a similar effect also occurred in the native agarose gel buffered at pH 8.3 leading to the nucleic acid being trapped in the wells.^23^ The plots of the frequency distributions and the average hydrodynamic diameter and percentage of polydispersity of the VNPs incubated at different pH for two hours are shown in Fig. 2BI and Table 2, respectively: at pH 4 VNPs maintained the same size as the control; however, a gradual increase in size and polydispersity was recorded at gradually lower pH of incubation. In other experiments CPMV was exposed to solutions at the same pH, but the pH was neutralised just prior to the DLS measurements (Fig. 2BII – Table 2). When CPMV was incubated at pH 3 and 4, the pH neutralisation returned CPMV to its exact native size; upon exposure of CPMV to pH 1.2 and 2 followed by pH neutralisation, aggregates of broad particle size distribution were detected. This is in agreement with the native agarose gel data.

The results suggest that following oral administration, CPMV would remain stable in fed gastric conditions (*i.e.* pH 3–7^36^), but might be unstable in fasting gastric conditions (*i.e.* pH 1–2^36^), leading to protein aggregation as the pH increases in the intestine.

### 3.2. Stability of CPMV upon exposure to simulated gastric fluids (SGF) and to simulated intestinal fluids (SIF) with digestive enzymes

**Fig. 3:**
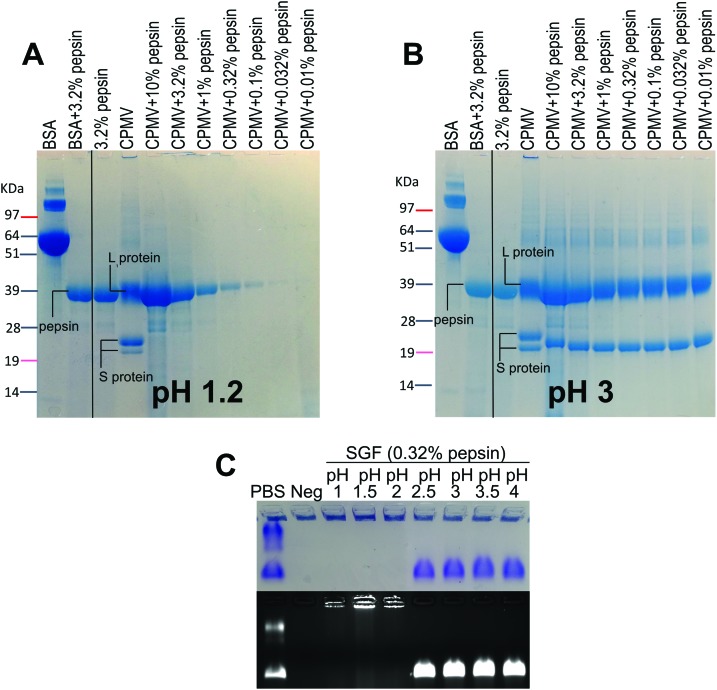
Chemical and physical stability of CPMV in simulated gastric fluids with pepsin. A and B: SDS-PAGE of CPMV incubated for 2 hours in SGF containing different concentrations of pepsin at pH 1.2 (A) or at pH 3 (B). CPMV (in water) and 3.2% pepsin were used as positive and negative controls, respectively. BSA was used as control to verify the enzymatic activity. C: Coomassie-stained (top) and ethidium bromide-stained (bottom) native agarose gel of CPMV after 2 hours incubation in SGF at different pH and containing fixed concentration of pepsin [0.32% (w/v)]. CPMV (in PBS) and SGF were used as positive and negative controls, respectively.

VNPs are protein-based NPs, therefore, they could potentially be digested by the proteolytic enzymes of the GI tract, *i.e.* pepsin in the stomach and the pancreatic proteases in the intestine.^20^Fig. 3 shows the effect of exposure to pepsin on the stability of CPMV. The stability tests were carried at the standard pepsin concentration in the SGF,^27^ but also at higher and lower pepsin concentrations, in order to reflect the extremely variable concentration of enzyme within the human stomach.^36^,^38^ In addition, the tests were performed at different pH, in order to assess the effect of pH on the enzymatic proteolysis of CPMV. Fig. 3A shows that at pH 1.2 both bovine serum albumin (BSA), used as control, and CPMV were digested after two hours exposure to pepsin, with the L and S proteins being undetectable at all pepsin concentrations. Fig. 3B shows that at pH 3, the 39 kDa protein band corresponding to the L protein remained present at all concentrations of enzyme, while the 25 kDa and 22 kDa S proteins were digested to give a single protein band of approximately 22 kDa. In stark contrast, the BSA control was totally digested. This indicates that CPMV is resistant to pepsin digestion at pH 3, but not at pH 1.2. In order to find the threshold pH at which CPMV became sensitive to the pepsin digestion, the VNPs were also incubated for two hours with various media at fixed pepsin concentration and variable pH (Table 1) and then analysed by electrophoresis on native agarose gels. The results, shown in Fig. 3C, revealed that the VNPs’ capsid remained stable when exposed to pepsin at pH ≥ 2.5, but were degraded at lower pHs.

Therefore, it can be concluded that CPMV sensitivity to pepsin digestion depends on the pH: at low pH, capsid destabilisation might render the L and S proteins susceptible to pepsin. By contrast, at pH ≥ 2.5, the highly compact viral structure remained intact (Fig. 2) shielding the protein from digestion. Partial digestion of the S protein into a slightly shorter peptide (22 kDa) at pH 3 (Fig. 3B) did not affect the particle stability, as shown by the native agarose gel (Fig. 3C). This is in agreement with previous published work, which showed that the C-terminus of the S protein of CPMV can be easily proteolytically cleaved without impacting the capsid stability.^37^

In the broader context of the oral delivery of NPs, these results suggests that VNPs are not likely to be digested by the pepsin in the stomach, unless the pH is low enough to destabilise the physical structure of the virus. Thus, although VNPs are protein-based NPs, their compact structure renders them much more resistant to digestion than globular proteins, such as BSA (Fig. 3B). This is in accordance with Wang *et al.* who have found that peptides with more rigid structures are less vulnerable to enzymatic cleavage in gastric and intestinal fluids than peptides with higher structural flexibility.^27^

The intestinal fluids could constitute another barrier to the oral delivery of VNP-based delivery systems, as pancreatic enzymes such as trypsin, chymotrypsin and elastase could potentially digest the polypeptides.^39^ As shown in Fig. SI1† particles remained intact upon exposure to SIF, in agreement with previous findings.^24^

### 3.3. Stability of CPMV upon exposure to pig gastric fluids (PGF)

Recently, efforts have been made to define suitable *in vitro* and *ex vivo* models for the prediction of the stability of biopharmaceuticals in human gastric and intestinal fluids:^26^ Wang *et al.* found a good correlation in the half-lives of 13 peptides in pig and human gastric fluids, indicating that pig gastric fluids are an effective tool to mimic the stability of protein-based biopharmaceuticals in human gastric fluids.^27^ CPMV was incubated in pig gastric fluids (PGF) to predict the fate of orally administered CPMV in humans. Possible barriers to the stability of VNPs in gastric fluids are: (1) proteolysis by the gastric enzymes;^23^ (2) pH-induced denaturation (Fig. 1 and 2); (3) aggregation;^40^ (4) formation of a protein corona around the particles, which could in turn affect size and surface properties and thus “screen” the biological properties and interactions of the VNPs *in vivo*.^22^,^41^

**Fig. 4:**
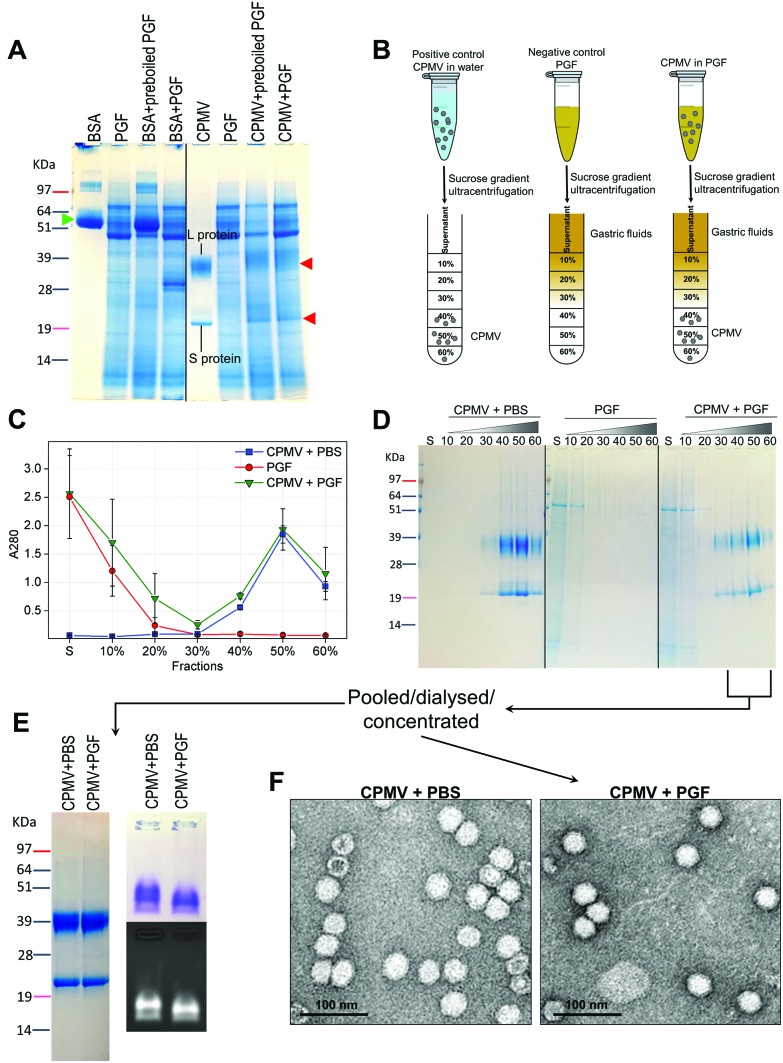
Chemical and physical stability of CPMV upon exposure to pig gastric fluids. A: SDS-PAGE of CPMV incubated in PGF for 2 hours. CPMV (in water) and PGF were used as positive and negative controls, respectively. BSA (green arrow) was used as control to verify the enzymatic activity. BSA and CPMV samples were also added to pre-boiled, *i.e.* inactivated, PGF for comparison purposes. L and S protein are indicated by red arrows. B: Schematic diagram of the sucrose gradient ultracentrifugation performed to separate CPMV from PGF. C: UV absorbance (mean ± SD, *n* = 3) at 280 nm of the sucrose gradient fractions of PGF, or of CPMV incubated for 2 hours in either PBS or PGF. D: SDS-PAGE of the same sucrose gradient fractions analysed in C; S, indicates the supernatant; 10, 20, 30, 40, 50 and 60 indicate the percentages of sucrose of the collected fractions. E and F: fractions of the gradient containing CPMV were pooled together, dialysed and concentrated and then analysed by SDS-PAGE (E left), native agarose gel (E right) and TEM (F).

Fig. 4 illustrates the chemical and physical stability of CPMV upon exposure to PGF for two hours. Fig. 4A shows the SDS-PAGE of samples of CPMV incubated in PGF: the BSA control was digested by the incubation in PGF, but it remained stable in pre-boiled, inactivated, PGF. By contrast, when CPMV was exposed to PGF, both the L and S proteins (red arrows) could still be visualised both in the inactive and active gastric media (Fig. 4A). To further characterise the stability of CPMV in PGF, after two hours incubation the pH was neutralised and the samples loaded on the top of a sucrose gradient. CPMV in PBS and PGF alone were used as positive and negative controls, respectively (Fig. 4B). After ultracentrifugation the fractions were collected for analysis of UV absorbance at 280 nm (A280) (Fig. 4C) and SDS-PAGE (Fig. 4D). In the PBS positive control, A280 was mainly detected in the 40, 50 and 60% bands; in the PGF negative control, high A280 was detected in the supernatant (due to the gastric protein content) and it gradually declined thereafter; in the sample where CPMV was incubated with PGF, high A280 was measured in the supernatant, it decreased gradually in the 10, 20 and 30% fractions, as in the negative control, and it increased again in the 40, 50 and 60% fractions as in the positive control, corresponding to CPMV (Fig. 4C). This indicates that sucrose gradient ultracentrifugation can efficiently separate CPMV from the gastric juice matrix, which remained in the upper fractions of the gradients. As CPMV was found in the same fractions as the positive control, the VNPs remain intact and neither denatured nor aggregated.^22^,^23^ Moreover, the A260/A280 ratio (data not shown), which is a marker of purity of VNPs preparations,^42^ was similar in the 40, 50 and 60% fractions of the positive control and in the sample where CPMV and PGF had been incubated (data not shown), thus indicating that the residual CPMV remaining after incubation in PGF and ultracentrifugation was not contaminated with other proteins.

Fig. 4D shows the SDS-PAGE analysis of the same fractions: after incubation of CPMV with PGF and sucrose density gradient ultracentrifugation, the proteins of the gastric fluids remained confined in the supernatant, 10 and 20% sucrose fractions (as in the PGF negative control), while CPMV L and S proteins banded in the 40, 50 and 60% fractions (as in the PBS positive control). This demonstrates that CPMV L and S proteins were not digested. The finding that VNPs exposed to PGF banded in exactly the same fractions as the positive control (Fig. 4C and D), suggests that their size and density did not change and indicates that CPMV was neither denatured, nor aggregated. This also provided a first suggestion that a protein corona adsorbed on the particle surface did not form. If a protein corona had formed and induced the formation of low density multi-particle agglomerates, these would have accumulated in the upper fractions of the gradient compared to pristine NPs, as reported in the case of magnetite NPs by Di Silvio *et al.*^22^

In order to further investigate stability, aggregation and evaluation of the protein corona, the fractions from the gradient containing CPMV were pooled together, dialysed and concentrated and then analysed by SDS-PAGE, native agarose gel (Fig. 4E) and TEM (Fig. 4F). Fig. 4E (left side) shows that the only proteins detectable in the CPMV preparation exposed to PGF, after ultracentrifugation, were the L and S proteins, consistent with a protein corona not forming. Fig. 4E (right side) shows that the electrophoretic migration of the VNPs in native agarose gel was only minimally affected by the incubation in PGF, indicating that the particles were intact and that their size and surface charge remained practically unchanged (indicating again no formation of a protein corona). Fig. 4F illustrates TEM imaging of the same preparations: VNPs exposed to PGF show the same icosahedral shape and size as the pristine VNPs. Furthermore, a protein corona cannot be visualised around the NPs. Indeed, the measured average diameter of the particles was similar for the pristine VNPs and those exposed to PGF (Table 3).

**Table 3:** Average particle diameter of CPMV and eVLPs incubated for two or four hours in PGF or PIF, respectively, as measured from TEM images (mean ± SD, *n* = 25)

	CPMV + PBS	CPMV + PGF	CPMV + PIF	eVLPs + PBS	eVLPs + PGF	eVLPs + PIF
Diameter (nm)	34.3 (±4.0)	33.3 (±1.8)	35.2 (±3.9)	33.2 (±2.1)	33.5 (±2.1)	35.7 (±2.9)

It must be pointed out that CPMV was stable in pig fluids that had pH = 3.9 (±0.1) (Table 1); while human gastric fluids, particularly if in fasting conditions, can have lower pH.^43^ Hence, CPMV might not withstand the fasting human gastric conditions, as suggested by Fig. 1–3.

### 3.4. Stability of CPMV upon exposure to pig intestinal fluids (PIF)

Similarly to PGF, pig intestinal fluids (PIF) can be used as surrogate of human intestinal fluids in the evaluation of the stability of biopharmaceuticals.^44^

**Fig. 5:**
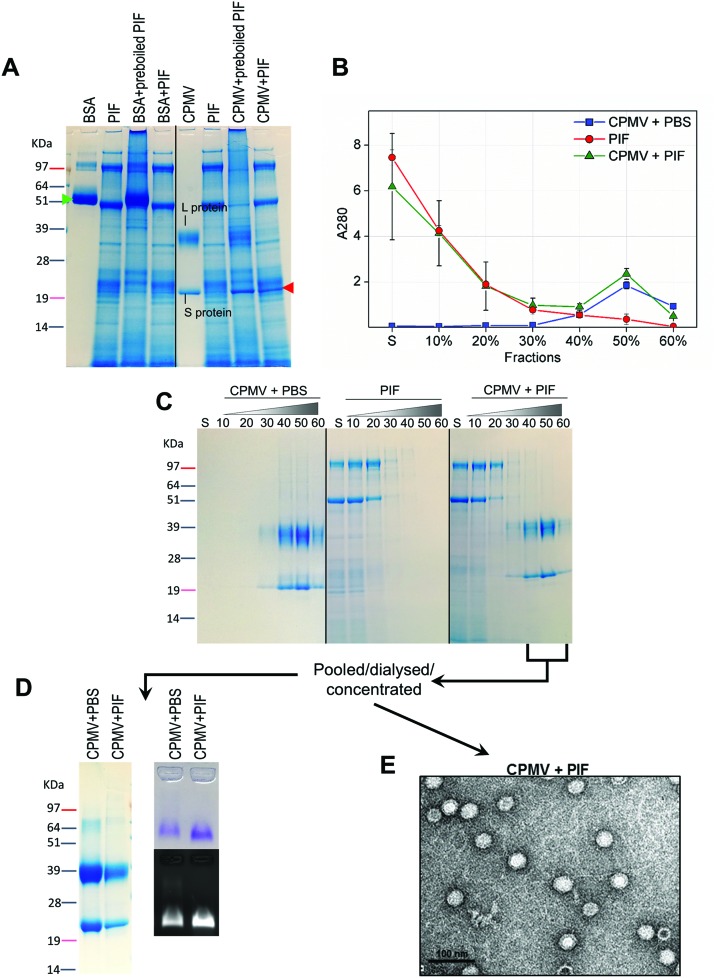
Chemical and physical stability of CPMV upon exposure to pig intestinal fluids. A: SDS-PAGE of CPMV incubated in PGF for 4 hours. CPMV (in water) and PIF were used as positive and negative controls, respectively. BSA was used as control to verify the enzymatic activity. BSA and CPMV samples were also added to pre-boiled, *i.e.* inactivated, PIF for comparison purposes. S protein is indicated by a red arrow. B: UV absorbance (mean ± SD, *n* = 3) at 280 nm of the sucrose gradient fractions of PIF or of CPMV incubated for 4 hours in PBS or PIF. C: SDS-PAGE of the same sucrose gradient fractions analysed in B; S, indicates the supernatant; 10, 20, 30, 40, 50 and 60 indicate the percentages of sucrose of the collected fractions. D and E: fractions of the gradient containing CPMV were pooled together, dialysed and concentrated and then analysed by SDS-PAGE (D left), native agarose gel (D right) and TEM (E).

For the assessment of stability of CPMV in PIF, similar experiments as those described for PGF were performed. However, in this case the VNPs were exposed to PIF for four hours, as this is the average intestinal transit time of pharmaceuticals.^45^ The SDS-PAGE gel (Fig. 5A) shows that while BSA control was digested in PIF, CPMV L protein remained intact (red arrow), although the S protein could not be clearly distinguished amongst all the proteins from the intestinal fluid. Using sucrose gradient ultracentrifugation, the separation of CPMV from the intestinal fluid matrix was possible (Fig. 5B): UV measurements of the sucrose fractions revealed that upon incubation of CPMV in PIF, protein could still sediment in the 40, 50 and 60% fractions of the gradient, as in the control in PBS. SDS-PAGE of the same sucrose fractions confirmed that the L and S proteins migrated to the same sucrose fractions for CPMV in PBS (control) and in PIF (Fig. 5C), thus indicating that the CPMV polypeptides were not digested and that NPs remained intact and not aggregated.

CPMV-rich gradient fractions, resulting from the incubation with PIF were pooled together, dialysed, concentrated and further analysed. No proteins apart from the L and S proteins were detected by SDS-PAGE (Fig. 5D); also the incubation in PIF did not affect the electrophoretic migration of CPMV in native agarose gel (Fig. 5D), signifying that size and surface charge remained unchanged. Finally, TEM images (Fig. 5E) and resulting average VNPs’ diameters (Table 3) showed no differences with respect to the pristine VNPs (Fig. 4F). All these findings indicated that, despite the protein-rich composition of the PIF, a protein corona did not form on the surface of the VNPs. These results are in agreement with the work of Pitek *et al.*^46^ who have shown that the total quantity of protein corona forming on the surface of tobacco mosaic virus-based VNPs upon exposure to human plasma was approximately 6 times less than that of synthetic NPs.

Similar to the incubation of CPMV in PGF, CPMV remained stable, dispersed as discrete particles and an evident protein corona could not be detected by any of the characterisation techniques used. It has been suggested that the pathway through which CPMV can reach the systemic circulation upon oral gavage in mice is by crossing the M cells covering the Peyer's patches lymphoid tissue in the small intestine.^25^ Therefore, these stable, protein corona-free VNPs could find applications as carriers for oral vaccines. In addition, it can be proposed that, given its ability to cross the small intestinal fluids unhindered, CPMV could also be used as a carrier for oral vaccines aiming to target the large-intestine: colonic vaccination has shown tremendous potential in protecting against genitorectal infections.^47^

A further hypothesis on future applications of CPMV in oral delivery can be proposed. It has been recently shown that several plant viruses are present in the human gut virome, not as pathogens, but as “commensals”. These plant viruses are probably introduced into the gut by the diet and remain viable: in fact a suspension of human faecal matter containing pepper mild mottle virus could still infect plants.^48^ It has been suggested that these plant viruses might have a role in modulating the qualitative and quantitative composition of the intestinal bacterial microbiota.^49^,^50^ Thus, one could propose that the direct oral administration of intestinally-stable plant-viruses, such as CPMV, could be exploited in the future as a means to regulate the intestinal microbiota.

### 3.5. Stability of eVLPs in simulated gastric fluids (SGF), simulated intestinal fluids (SIF), pig gastric fluids (PGF) and pig intestinal fluids (PIF)

From a drug delivery point of view, empty virus-like particles (eVLPs) of CPMV, devoid of nucleic acid could have advantages over CPMV, including reduced bio-safety concerns and more efficient bio-conjugation of molecules at the interior surface of particles.^4^,^9^ From an oral delivery perspective, it is not known whether the structural difference between hollow eVLPs and filled CPMV could have an influence on their stability in simulated and pig GI fluids.

**Fig. 6:**
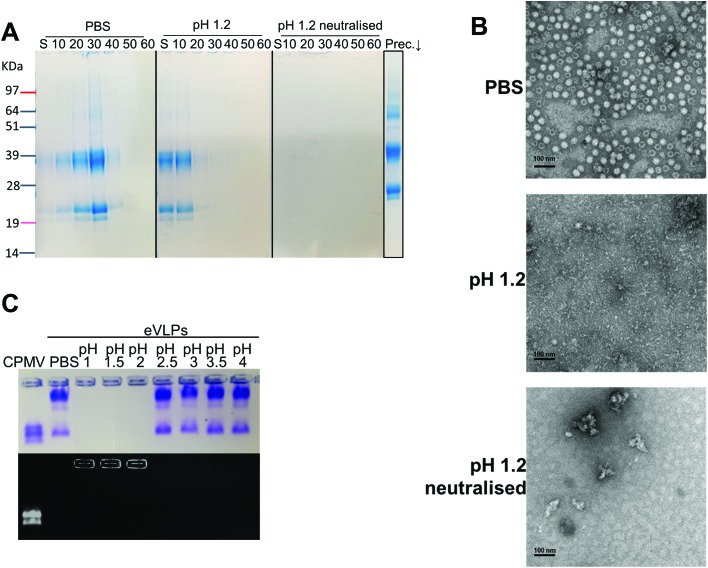
Physical stability of eVLPs in simulated gastric fluids at different pH (without pepsin). A: SDS-PAGE of sucrose gradient fractions, after 2 hours incubation of eVLPs in PBS (control), pH 1.2 and pH 1.2 followed by pH neutralisation. S, indicates the supernatant; 10, 20, 30, 40, 50 and 60 indicate the percentages of sucrose of the collected fractions; Prec.↓ indicates the precipitate formed after the ultracentrifugation. B: TEM images of eVLPs after 2 hours incubation in PBS (control), pH 1.2 and pH 1.2 followed by pH neutralisation; (scale bar = 100 nm). C: Coomassie-stained (top) and ethidium bromide-stained (bottom) native agarose gel of eVLPs after 2 hours incubation in simulated gastric fluids at different pH and in PBS (control). CPMV was also used as control.

Initially, eVLPs were incubated in PBS (positive control), pH 1.2 or pH 1.2 neutralised and analysed by sucrose density gradient ultracentrifugation followed by SDS-PAGE analysis of the fractions, as shown in Fig. 6A. It can be noticed that the L and S protein sediment mainly in 10, 20 and 30% fractions of the gradient, higher up compared to CPMV, using identical experimental conditions (Fig. 1). This is expected, because, although the capsid structure of CPMV and eVLPs is identical, eVLPs are less dense, as they are devoid of nucleic acid.^51^ At pH 1.2 the L and S proteins banded mainly in the supernatant and in the 10% sucrose fractions, indicating that the capsid disassembled and the polypeptides denatured. At pH 1.2, followed by neutralisation, the L and S proteins could be found as a precipitate at the bottom. TEM images (Fig. 6B) confirmed that the eVLPs had disassembled at pH 1.2 and the L and S proteins had aggregated when the pH was neutralised.

A comparison between Fig. 1B and C and 6A and B, reveals that while CPMV incubated at pH 1.2 could still penetrate the 10, 20, 30 and 40% gradient fractions (Fig. 1B) and could still be visualised as particles (Fig. 1C), eVLPs in the same conditions did not sediment in the gradient and were totally denatured (Fig. 6A and B). These results do not necessarily mean that CPMV were not denatured: indeed, Da Poian *et al.* showed that when CPMV and eVLPs had been exposed to high pressure, eVLPs disassembled whilst the protein–protein contact was broken in CPMV but the protein remained bound to the nucleic acid core.^52^ Similarly, in our study, it is possible that protein denaturation occurred for eVLP and also for CPMV exposed to pH 1.2, but CPMV still appeared as particles, due to the partially denatured protein remained in close proximity to the nucleic acid. The observation that, when the pH was raised to neutrality both CPMV and eVLPs aggregated, is a further indication that the protein structure had been previously (during the incubation in acid) irreversibly altered, *i.e.* denatured, in both types of VNPs preparations.

As for CPMV, native agarose gel showed that the eVLPs were stable at pH ≥ 2.5 (Fig. 6C) and unstable at lower pH.

The stability and aggregation of eVLPs in SGF, SIF, PGF and PIF were similar to those described for CPMV and are shown in Fig. SI2–SI5,† respectively. The average diameter of the eVLPs after incubation in PGF and PIF was also the same as that of pristine NPs (Table 3), indicating that the eVLPs were stable and did not form a protein corona, as also observed for CPMV. This is expected as CPMV and eVLPs share the same surface properties.^51^,^53^

## 4. Conclusions

In the last few years, plant-based viruses have emerged as a new and promising class of nanotechnology systems. However, to date the use of VNPs in oral drug delivery has been only minimally explored. The results of this study suggest that upon oral administration, CPMV-based NPs are likely to remain stable in the stomach in the fed condition and in the intestine; however, the NPs would be denatured and digested in fasting gastric conditions where the pH ranges from 1 to 2.^36^ Also, upon oral administration VNPs are likely to maintain their native corona-free structure in the GI fluids, and therefore specific and non-specific biological interactions of these NPs *in vivo* (including passive and active targeting) would remain predictable and controllable. While for most synthetic NPs, protein corona formation can be reduced by conjugation of polyethylene glycol (PEG) or other molecules at the surface of NPs,^41^ a similar approach would be unnecessary for VNPs as even in their native state they should remain clear from adsorbed protein in biological fluids.

In the broader context, VNPs should be ideal nanotechnology systems for oral drug delivery, as in many aspects they can outmatch other existing nanomaterials. However, a simple gastro-resistant formulation is likely to be needed to shield VNPs from the fasting conditions of the stomach, which has lower pH than that of the pig fluids tested in this study.^43^

## Conflicts of interest

G.P.L. declares that he is a named inventor on granted patent WO 29087391 A1 which describes the transient expression system used in this manuscript.

## Supplementary Material

Supplementary information
