# Supplementary material for: Stability of plant virus-based nanocarriers in gastrointestinal fluids
Source: Nanoscale. 2017 Dec 5;10(4):1667–79. doi: 10.1039/c7nr07182e (PMC5804478; doi:10.1039/c7nr07182e)
Supplement: Supplementary file 1 [file NR-010-C7NR07182E-s001.pdf]

Electronic Supplementary Material (ESI) for Nanoscale.
This journal is © The Royal Society of Chemistry 2017

Supplementary Information

Figure SI1

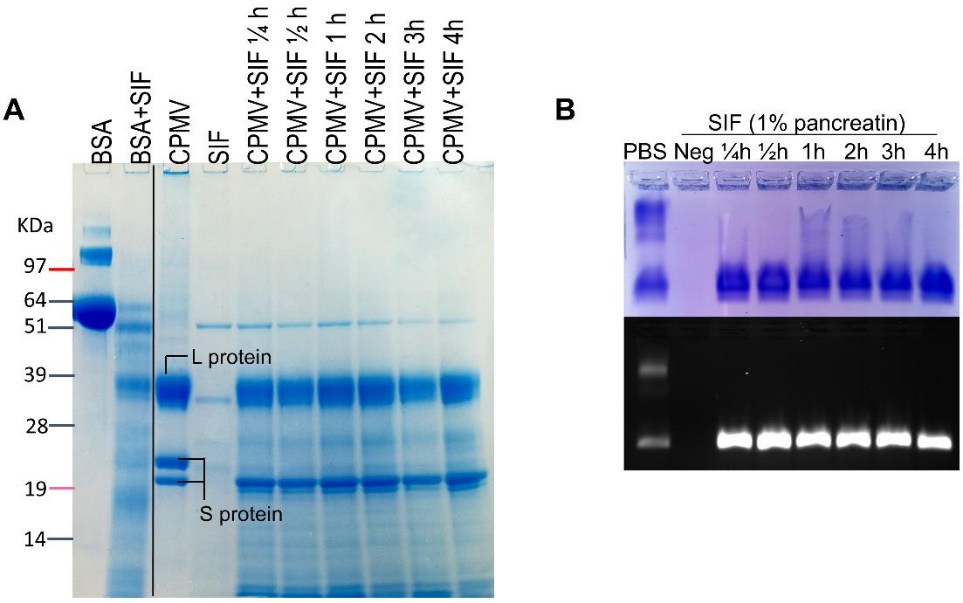

Figure SI1: Chemical and physical stability of CPMV in simulated intestinal fluids. A: SDS-PAGE of CPMV incubated for different intervals of time in SIF. CPMV (in water) and SIF were used as positive and negative controls, respectively. BSA was used as control to verify the enzymatic activity. B: Coomassie-stained (top) and ethidium bromide-stained (bottom) native agarose gel of CPMV after incubation in SIF. CPMV (in PBS) and SIF were used as positive and negative controls, respectively. In can be noticed that in the native agarose gel two bands are present in the control in PBS, but, only the lower, faster migrating, band remained when CPMV was incubated in the SIF. Sainsbury at al. found that the S protein naturally exists as both a short and long version and that two-to-three weeks of aging of the preparation or chymotrypsin treatment induces cleavage of 24 amino acids at the C-terminus of the long form, thus converting all the S protein into the short form; this cleavage does not have any impact on the integrity of the particles, which are visualised as the faster migrating band in the native agarose gels [1]. The digestion pattern described is the same as that seen in this Figure.

Figure SI2

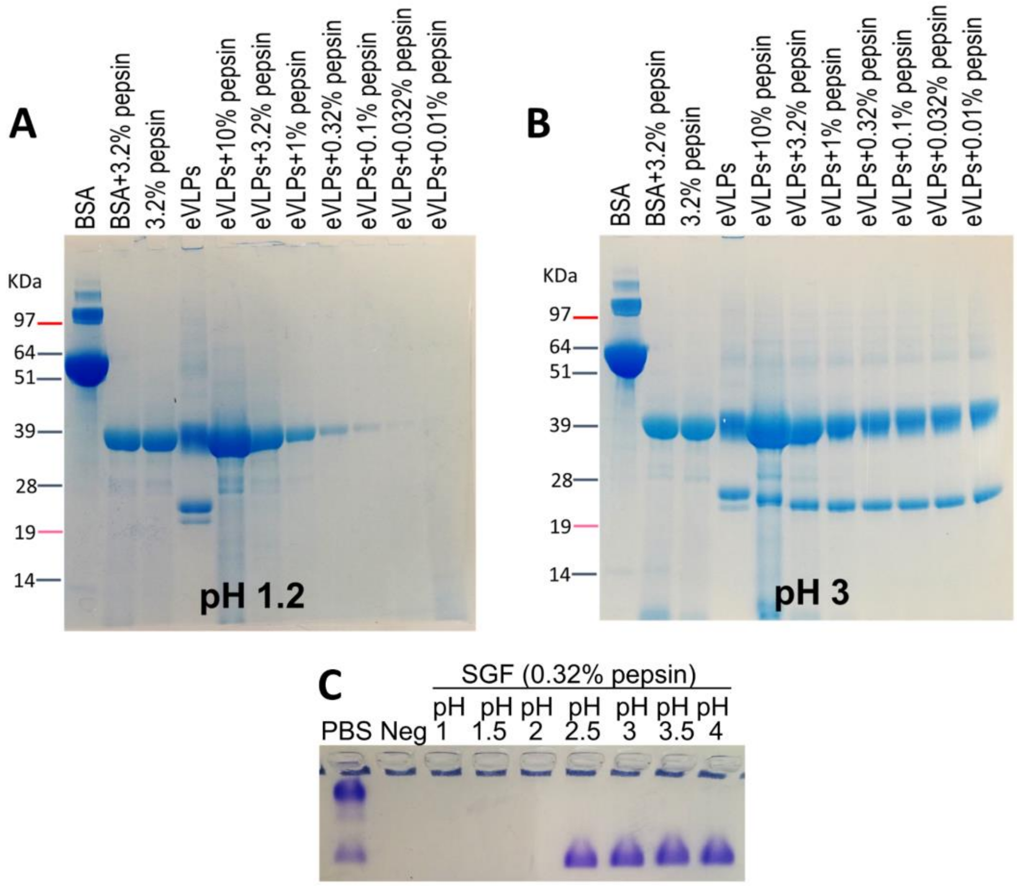

Figure SI2: Chemical and physical stability of eVLPs in simulated gastric fluids with pepsin. A and B: SDS-PAGE of eVLPs incubated for 2 hours in SGF containing different concentrations of pepsin at pH 1.2 (A) or at pH 3 (B). eVLPs (in water) and 3.2% pepsin were used as positive and negative controls, respectively. BSA was used as control to verify the enzymatic activity. C: Coomassie-stained native agarose gel of eVLPs after 2 hours incubation in SGF at different pH and containing fixed concentration of pepsin [0.32% (w/v)]. eVLPs (in water) and SGF were used as positive and negative controls, respectively.

Figure SI3

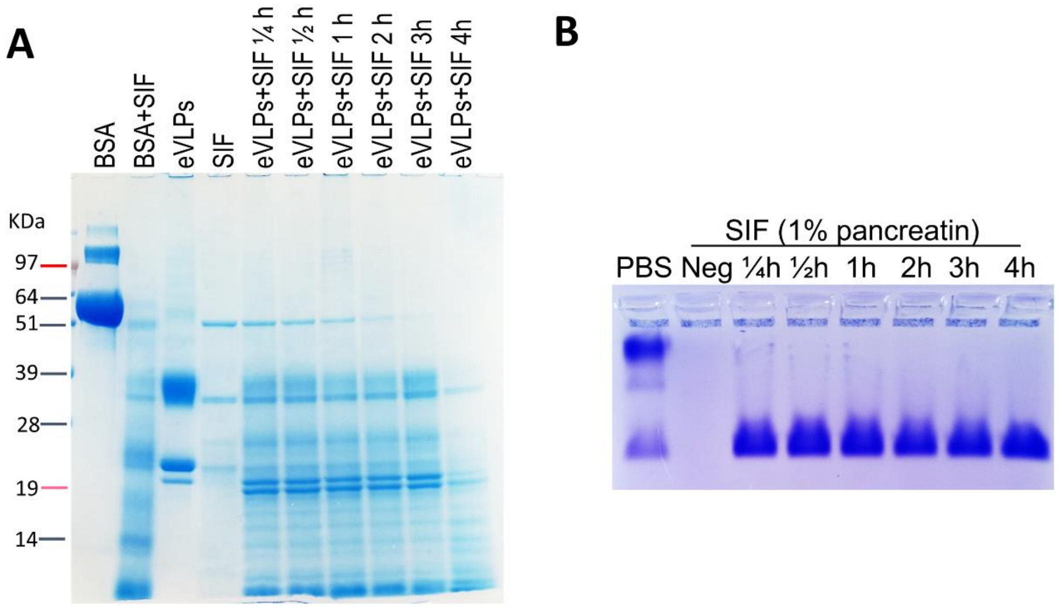

Figure SI3: Chemical and physical stability of eVLPs in simulated intestinal fluids. A: SDS-PAGE of eVLPs incubated for different intervals of time in SIF. eVLPs (in water) and SIF were used as positive and negative controls, respectively. BSA was used as control to verify the enzymatic activity. B: Coomassie-stained native agarose gel of CPMV after incubation in SIF. CPMV (in water) and SIF were used as positive and negative controls, respectively.

Figure SI4

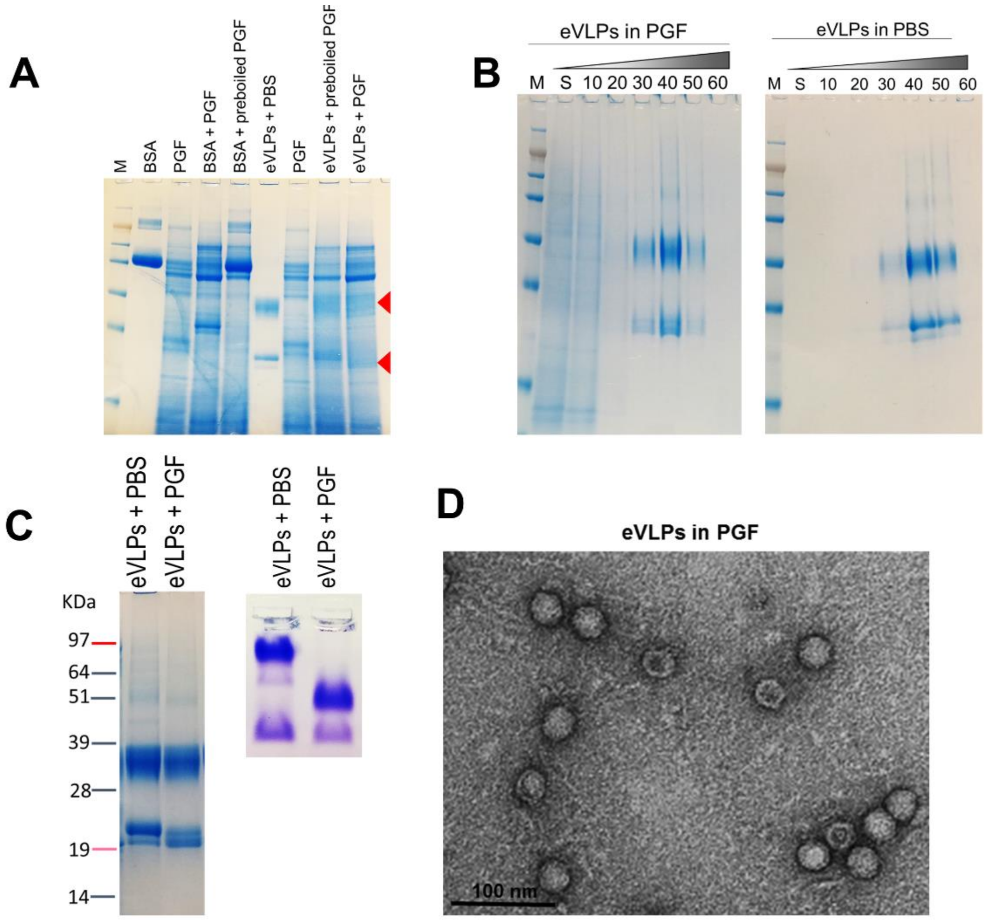

Figure SI4: Chemical and physical stability of eVLPs upon exposure to pig gastric fluids. A: SDS-PAGE of eVLPs incubated in PGF for 2 hours. eVLPs (in water) and PGF were used as positive and negative controls, respectively. BSA was used as control to verify the enzymatic activity. BSA and eVLP samples were also added to pre-boiled, i.e. inactivated, PGF for comparison purposes. L and S protein are indicated by red arrows. B: sucrose gradient ultracentrifugation was performed to separate eVLPs from PGF. SDS-PAGE of the sucrose gradient fractions of eVLPs incubated for 2 hours in PGF or in PBS. C and D: SDS-PAGE (C left), native agarose gel (C right) and TEM (D) of dialysed and concentrated sucrose fractions containing eVLPs, previously incubated in PGF.

Figure SI5

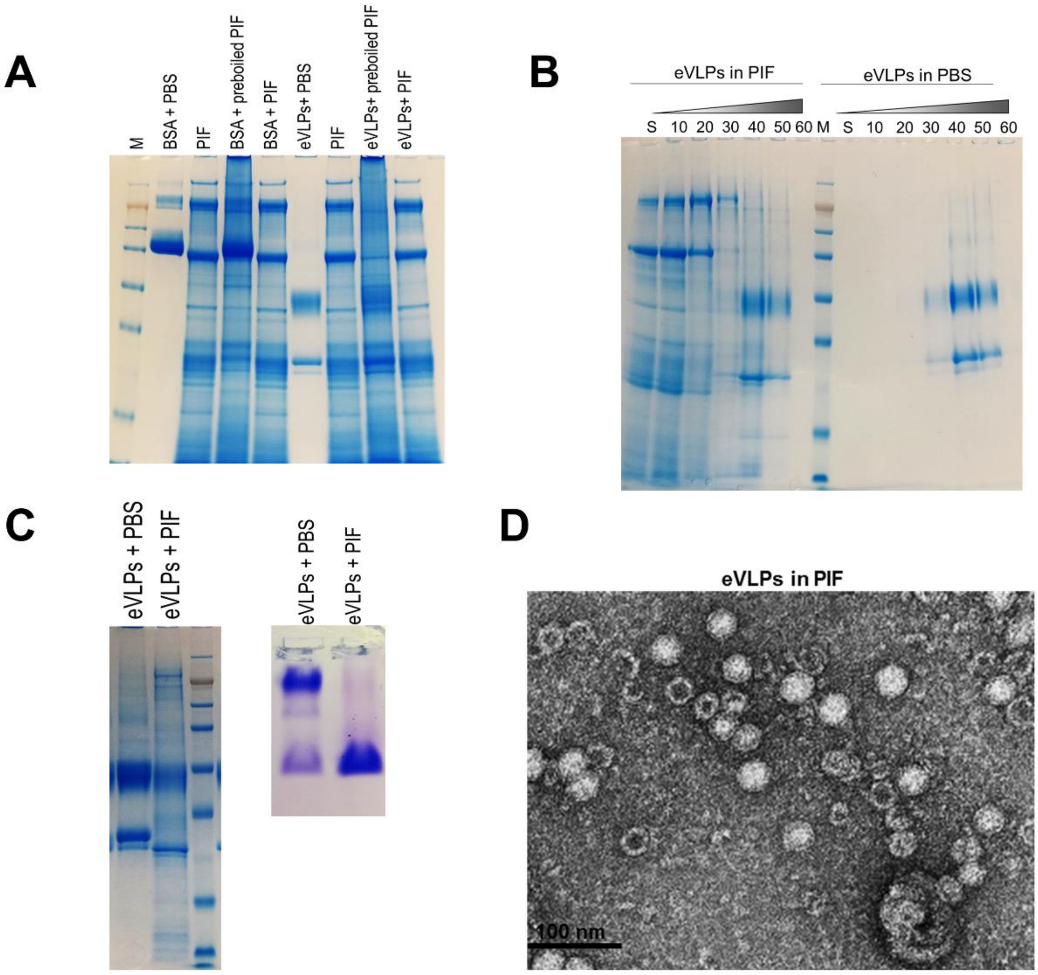

Figure SI5: Chemical and physical stability of eVLPs upon exposure to pig intestinal fluids. A: SDS-PAGE of eVLPs incubated in PIF for 4 hours. eVLPs (in water) and PGF were used as positive and negative controls, respectively. BSA was used as control to verify the enzymatic activity. BSA and eVLP samples were also added to pre-boiled, i.e. inactivated, PIF for comparison purposes. B: sucrose gradient ultracentrifugation was performed to separate eVLPs from PIF. SDS-PAGE of the sucrose gradient fractions of eVLPs incubated for 4 hours in PIF or in PBS. C and D: SDS-PAGE (C left), native agarose gel (C right) and TEM (D) of dialysed and concentrated sucrose fractions containing eVLPs, previously incubated in PIF. Protein other than the L and S protein can be visualised in the SDS-PAGE (C left). The presence of this protein is due to impurity of the sucrose gradient fractions pooled together, dialysed, concentrated and then analysed by SDS-PAGE. Indeed, as eVLPs are less dense than CPMV they do not sediment as low in the gradient as CPMV, the separation from PIF by gradient ultracentrifugation could not be as efficient. However, native agarose gel (C right), TEM (D) and the measurement of the eVLPs' diameter (Table 3) demonstrated that the protein present in the sample is not protein corona, but globular impurities of PIF that co-migrated in the same gradient fractions as the eVLPs.

Reference

1 F. Sainsbury, K. Saunders, A. A. A. Aljabali, D. J. Evans and G. P. Lomonossoff, *ChemBioChem*, 2011, **12**, 2435–2440.
